# Keratoconus tomographic indices in osteogenesis imperfecta

**DOI:** 10.1007/s00417-023-06059-4

**Published:** 2023-04-19

**Authors:** Rafael Correia Barão, Miguel Santos, Raquel Esteves Marques, Ana Miguel Quintas, Paulo Guerra

**Affiliations:** 1 Department of Ophthalmology, Hospital de Santa Maria, CHULN, Av. Prof. Egas Moniz, 1649-035 Lisbon, Portugal; 2 Visual Sciences Study Center, Faculty of Medicine, University of Lisbon, Lisbon, Portugal

**Keywords:** Corneal tomography, Keratoconus, Collagen, Osteogenesis

## Abstract

**Purpose:**

Osteogenesis imperfecta (OI) is a rare inherited disease affecting collagen-rich tissues. Ocular complications have been reported such as thin corneas, low ocular rigidity, keratoconus, among others. The purpose of this study is to characterize corneal tomographic features in OI patients compared to unaffected patients, with particular focus on commonly studied keratoconus indices.

**Methods:**

Cross-sectional case–control study including 37 OI patients and 37 age-matched controls. Patients and controls underwent comprehensive ophthalmological examination including corneal Scheimpflug tomography with a Pentacam HR device (Oculus Optikgeräte GmbH, Wetzlar, Germany) to analyse and compare topometric, tomographic, pachymetric and Belin-Ambrósio Enhanced Ectasia Display III (BAD-D) data of both eyes of each patient.

**Results:**

Most OI patients had type I disease (*n* = 24; 65%) but type III–VII patients were also included. Two patients had clinically overt bilateral keratoconus. OI patients had significantly higher maximum keratometry (45.2 ± 2.1 vs. 43.7 ± 1.2; *p* = 0.0416), front and back elevation (3.0 ± 3.3 vs. 2.1 ± 1.3, p = 0.0201; 11.1 ± 8.2 vs. 5.0 ± 3.7, *p* < 0.0001), index of surface variance (25.5 ± 13 vs. 17.4 ± 8.3; *p* = 0.0016), index of vertical asymmetry (0.21 ± 0.14 vs. 0.15 ± 0.06; *p* = 0.0215), index of height asymmetry (9.2 ± 14 vs. 6.0 ± 4.5; *p* = 0.0421), index of height decentration (0.02 ± 0.01 vs. 0.01 ± 0.01; *p* < 0.0001) and average pachymetric progression (1.01 ± 0.19 vs. 0.88 ± 0.14;* p* < 0.0001) readings. Thinnest corneal thickness and maximum Ambrósio relational thickness were significantly lower (477 ± 52 vs. 543 ± 26; 387 ± 95 vs. 509 ± 49; *p* < 0.0001). Two-thirds of OI patients had corneas with a minimum thickness < 500 µm. BAD-D value was significantly higher in OI patients (2.1 ± 1.4 vs. 0.9 ± 0.2; *p* < 0.0001).

**Conclusion:**

OI patients showed significant changes in corneal profiles compared with healthy subjects. A high proportion of patients had tomographically suspect corneas when using keratoconus diagnostic indices. Further studies are warranted to assess the true risk of corneal ectasia in OI patients.


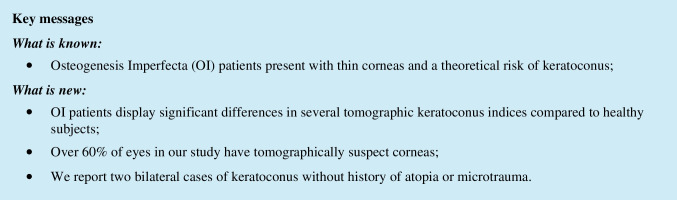


## Introduction

Osteogenesis imperfecta (OI) is a rare inherited connective tissue disease affecting collagen production, with a prevalence around 6–7:100,000, commonly caused by mutations in the COL1A1 and COL1A2 genes, which encode α1 and α2 chains of type I collagen respectively, with a predominantly autosomal dominant inheritance pattern [1]. Several clinical forms of OI have been described according to early lethality, onset and number of fractures, skeletal deformity, patient stature, radiological and other clinical findings. Classically, OI patients have been classified into OI types I–IV according to Sillence [1–3]. OI type I (“non-deforming OI with blue sclerae”), the most common and mildest clinical form, is generally caused by COL1A1 or COL1A2 mutations determining a quantitative deficit of collagen type I, with patients suffering multiple fractures during childhood up to late puberty. Type II OI is severe and almost always lethal perinatally. OI type III is associated a qualitative collagen defect, with more numerous and severe fractures in childhood than type I, progressive deformation, and a wider variety of causative genetic mutations. OI type IV (“common variable OI with normal sclerae”) is the second most prevalent type and displays a clinical picture similar to OI type I but several genetic loci have been implicated. With the advent of widespread genetic testing and wide-genome assessments, more genetic defects in different targets pertaining to collagen metabolism have been identified as causative, which have expanded the original Sillence classification to include novel and rarer OI forms recognised by the Online Mendelian Inheritance in Man (OMIM) database [4–7]. These rarer forms include OI type V (OMIM #610,967; autosomal dominant OI with calcification in interosseous membranes, caused by IFITM5 mutations), type VI (OMIM #613,982, autosomal recessive, similar to type IV but usually more severe), type VII (OMIM #610,682, autosomal recessive severe and progressively deforming), among others.

Patients with OI can display several multisystem manifestations. The classic ocular sign of OI is a blueish-grey discoloration of the sclerae, particularly in type I disease [8]. However, other ocular manifestations have been reported, such as absent or atrophic Bowman’s layer, keratoconus (KC), high myopia, glaucoma, low ocular rigidity, reduced corneal hysteresis, increased risk of scleral rupture, retinal haemorrhages, subretinal and choroidal neovascularization, among others [9–29]. Corneal thinning has been recently highlighted as a possible hallmark of the disease, particularly in OI type I [10, 24]. Most of these changes seem to empirically stem from a connective tissue defect and higher tissue fragility, given that the corneoscleral layer contains predominantly type I collagen [30–32]. However, the risk of these manifestations in OI patients has not been well studied and most associations arise from case reports and small series, including in the case of KC [9, 33, 34]. Although clinical KC diagnosis with direct observation at the slit-lamp is possible, particularly in advanced cases, corneal tomographic analysis is currently the screening, diagnostic and progression analysis tool of choice [35–37].

The present study aims to characterize corneal tomographic features in OI patients compared to unaffected patients, with particular focus on commonly used and clinically validated KC diagnostic indices.

## Materials and methods

### Design and population

A cross-sectional observational case–control study was undertaken from 2019 to 2022 in Hospital Santa Maria, CHULN, Lisbon, Portugal. Thirty-seven (37) Portuguese adult patients with OI diagnosis and 37 age-matched controls were included in this study. All patients included had an OI diagnosis confirmed by a medical genetics expert based on a compatible clinical, each with a lifelong history of multiple and recurring low trauma fractures and compatible radiological findings. Disease classification was made from the Skeletal Dysplasia Nomenclature Group [3] and the Online Mendelian Inheritance in Man (OMIM) database [6] according to clinical and genetic findings. Exclusion criteria were age < 18 years, uncertain diagnosis, previous corneal surgery or trauma. All control patients included were healthy subjects who came in for routine clinical observation and had normal ophthalmological examinations other than refractive error within 1.0 dioptre (D) of spherical equivalent. Approval was obtained from the CHULN/CAML ethics committee and this study adhered to the tenets of the Declaration of Helsinki.

### Ophthalmological evaluation

Patients underwent ophthalmological evaluation, including autorefraction, intraocular pressure (IOP) measurement using Goldmann applanation tonometry (GAT), slit-lamp biomicroscopy and mydriatic fundus examination. Patient and control eyes also underwent corneal Scheimpflug tomography using a Pentacam HR device (Oculus Optikgeräte GmbH, Wetzlar, Germany). Patients wearing contact lenses were instructed to discontinue its use before undergoing corneal tomography (for at least 2 weeks for rigid gas-permeable lenses and 48 h for soft lenses). Topographic, tomographic and pachymetric parameters included in this analysis included mean front and maximum keratometry (Km and Kmax respectively), net power of corneal astigmatism (Ast), thinnest corneal thickness (CTmin), anterior and posterior surface elevation at the thinnest point in relation to a 8-mm best-fit sphere (FEle and BEle respectively), index of surface variance (ISV), index of vertical asymmetry (IVA), index of height asymmetry (IHA), index of height decentration (IHD), average pachymetric progression index (PPIavg), maximum Ambrósio relational thickness (ARTmax) and Belin–Ambrósio Enhanced Ectasia Display III software-generated D score for deviation from normality (BAD-D). Pertinent clinical data was retrieved from patient files.

### Statistical analysis

This study was powered to detect a clinically relevant difference in BAD-D and thinnest corneal thickness. A minimum sample size of 34 subjects in each group would be required to detect a 1-point change in BAD-D and 50 µm in thinnest corneal thickness, assuming a mean ± standard deviation of 0.96 ± 0.8 for BAD-D and 539 ± 31 µm for corneal thickness, as reported in previous studies [35, 38], with a 2-sided significance level of 0.003 and 99% confidence level [39].

Statistical analysis was performed with GraphPad Prism 9 (version 9.5.0, San Diego, CA, USA). Kolmogorov–Smirnov and Shapiro–Wilk tests were used to assess distribution normality. Chi-square test was used for categorical variables, whereas Student *t* and Kolmogorov–Smirnov tests were used for parametric and non-parametric quantitative data analysis, respectively. Pearson and Spearman correlation analyses were performed between variables with a normal or non-normal distribution, respectively. A *p*-value < 0.05 was used to assert statistical significance.

## Results

Both eyes of thirty-seven (37) OI patients and 37 age-matched controls were included in this analysis. Age distribution was similar between OI and controls (*p* = 0.8467) as was female to male ratio (30:7 in the OI group and 29:8 in the control group). Of the 37 OI patients, 24 (65%) were classified as OI type I, most of whom had quantitative COL1A1 mutations (23/24). The remaining 13 patients had qualitative mutations and were classified as OI type III (*n* = 3, 8%), type IV (*n* = 7, 19%), type V (*n* = 1, 3%), type VI (*n* = 1, 3%) and type VII (*n* = 1, 3%). All OI type I and type III patients displayed blue sclerae bilaterally, a significantly higher proportion than in the type IV–VII subgroup (*n* = 27 vs. *n* = 1; *p* < 0.0001). All patients but one in each group (OI and controls) were phakic, and there was no other surgical history reported. Two OI type I patients had a previous diagnosis of bilateral keratoconus, with compatible clinical signs at the slit-lamp and used rigid gas-permeable contact lenses as a visual aid. No other patient used contact lenses. One OI patient had a history Behçet’s disease complicated with previous posterior uveitis and uveitic glaucoma under topical medication. No patient in either group (OI or control) had history of atopic disease such as allergies, asthma or eczema, or reported history of eye-rubbing behaviour or corneal microtrauma, including those with diagnosed KC. Regarding familiarity, patients 29 and 30 were siblings, and patients 3 and 2 were mother and son. No other familial relationship was recorded. Clinical classification, mutated genes and overall ocular findings in OI patients are detailed in Table 1.

**Table 1:** OI patient clinical classification, mutated gene and ocular findings

Patient #	Age	OI type [3, 6]	Mutated gene	Blue sclerae	Other findings
1	51	I	*COL1A1*	Yes	–
2	36	I	*COL1A1*	Yes	–
3	22	I	*COL1A1*	Yes	–
4	23	I	*COL1A1*	Yes	–
5	25	VI	*SERPINF1*	No	–
6	56	I	*COL1A1*	Yes	–
7	52	I	*COL1A1*	Yes	–
8	51	I	*COL1A1*	Yes	–
9	49	I	*COL1A1*	Yes	Bilateral KC
10	58	I	*COL1A2*	Yes	–
11	30	I	*COL1A1*	Yes	–
12	39	IV	*WNT1*	No	–
13	36	VII	*CRTAP*	No	–
14	23	I	*COL1A1*	Yes	Bilateral KC
15	30	IV	*SERPINF1*	No	–
16	25	IV	*COL1A1*	No	–
17	59	III	*COL1A1*	Yes	–
18	23	IV	*COL1A1*	No	–
19	64	I	*COL1A1*	Yes	–
20	49	IV	*COL1A1*	Yes	–
21	63	IV	*COL1A1*	No	–
22	57	I	*COL1A1*	Yes	Bilateral pseudophakia
23	35	I	*COL1A1*	Yes	–
24	44	V	*IFITM5*	No	–
25	77	I	*COL1A1*	Yes	–
26	53	I	*COL1A1*	Yes	–
27	44	I	*COL1A1*	Yes	–
28	53	III	*COL1A1*	No	–
29	23	I	*COL1A1*	Yes	Behçet’s disease, inactive posterior uveitis, glaucoma
30	32	I	*COL1A1*	Yes	–
31	45	I	*COL1A1*	Yes	–
32	45	I	*COL1A1*	Yes	–
33	36	I	*COL1A1*	Yes	–
34	18	I	*COL1A1*	Yes	–
35	23	I	*COL1A1*	Yes	–
36	46	III	Unknown	Yes	–
37	62	IV	Unknown	No	–

All comparisons except mean keratometry (Km, 43.6 ± 1.9 vs. 43.7 ± 1.2, *p* = 0.6330) showed significant differences between groups (Table 2). Spherical and cylindrical errors, true net corneal astigmatism power and Kmax were all higher in OI patients versus controls (1.6 ± 5.1 vs. 0.2 ± 0.5; 1.3 ± 0.9 vs. 0.4 ± 0.2; 1.5 ± 0.8 vs. 0.3 ± 0.2; 45.2 ± 2.1 vs. 43.7 ± 1.2; *p* < 0.05 for all comparisons). Elevation maps showed higher front and back elevation at thinnest point in OI patients (3.0 ± 3.3 vs. 2.1 ± 1.3, p = 0.0201; 11.1 ± 8.2 vs. 5.0 ± 3.7*, p* < 0.0001). ISV, IVA, IHA and IHD were also higher in OI patients (*p* < 0.05 for all; see Table 2). Pachymetric measurements such as thinnest corneal thickness and ARTmax were significantly lower (477 ± 52 vs. 543 ± 26; 387 ± 95 vs. 509 ± 49; *p* < 0.0001 for both) whereas PPIavg was higher in OI patients (1.01 ± 0.19 vs. 0.88 ± 0.14; *p* < 0.0001). The proportion of OI patient eyes with thinnest pachymetry < 500 µm was significantly higher than in controls (*n* = 49 vs. 2; 66.2% vs. 2.7%; *p* < 0.0001). The BAD final D value was significantly higher in OI patients (2.1 ± 1.4 vs. 0.9 ± 0.2; *p* < 0.0001).

**Table 2:** Age, IOP, refraction and Pentacam corneal tomography readings comparison between OI and control groups and between OI type I eyes and non-type I

Variable (m ± SD)	OI eyes (*n* = 74)	Control eyes (*n* = 74)	*p*-value OI vs. controls	OI type I eyes (*n* = 48)	OI types III-VII eyes (*n* = 26)	*p*-value OI type I vs. types III-VII
Age, years [range]	42 ± 15 [18–77]	41 ± 15 [19–75]	0.8467^#^	42 ± 16 [18–77]	43 ± 14 [23–63]	0.8725^#^
IOP, mmHg	13.9 ± 4.6	16.5 ± 5.0	< 0.0001*	13.2 ± 4.7	15.2 ± 4.2	0.1019*
Spherical error, D	−1.6 ± 5.1	0.2 ± 0.5	< 0.0001*	−1.0 ± 4.2	−0.9 ± 6.3	0.0939*
Cylindrical error, D	1.3 ± 0.9	0.4 ± 0.2	< 0.0001*	1.4 ± 0.9	0.9 ± 1.2	0.6538*
Ast, D	1.5 ± 0.8	0.3 ± 0.2	** < 0.0001**^**#**^	1.4 ± 0.8	1.6 ± 0.9	0.3120^#^
Km, D	43.6 ± 1.9	43.7 ± 1.2	0.6330^#^	43.6 ± 2.0	43.7 ± 1.6	0.8009^#^
Kmax, D	45.2 ± 2.1	45.5 ± 2.1	0.0416*	45.2 ± 2.3	45.2 ± 1.8	0.4974*
CTmin, µm	477 ± 52	543 ± 26	** < 0.0001**^**#**^	456 ± 40	517 ± 50	** < 0.0001**^**#**^
FEle, µm	3.0 ± 3.3	2.1 ± 1.3	0.0201*	3.5 ± 3.8	2.0 ± 1.8	0.1944*
BEle, µm	11.1 ± 8.2	5.0 ± 3.7	< 0.0001*	12.4 ± 9.1	8.6 ± 5.8	0.1496*
ISV	25.5 ± 13	17.4 ± 8.3	0.0016*	27.9 ± 15	21.2 ± 7.1	0.1541*
IVA	0.21 ± 0.14	0.15 ± 0.06	0.0215*	0.23 ± 0.15	0.17 ± 0.08	0.4669*
IHA	9.2 ± 14	6.0 ± 4.5	0.0421*	11 ± 17	5.6 ± 5.2	0.1635*
IHD	0.02 ± 0.01	0.01 ± 0.01	< 0.0001*	0.02 ± 0.02	0.01 ± 0.01	0.1784*
PPIavg	1.01 ± 0.19	0.88 ± 0.14	< 0.0001*	1.05 ± 0.18	0.95 ± 0.17	0.0936*
ARTmax	387 ± 95	509 ± 49	** < 0.0001**^**#**^	361 ± 85	435 ± 95	**0.0017**^**#**^
BAD-D	2.1 ± 1.4	0.9 ± 0.2	< 0.0001*	2.5 ± 1.5	1.3 ± 0.8	0.0055*

Bold entries are statistically significant

*Abbreviations*: *ARTmax* Ambrósio relational thickness, *Ast* true net corneal astigmatism power, *BAD-D* Belin-Ambrósio Enhanced Ectasia D value for deviation from normality, *CTmin* thinnest corneal thickness, *D* dioptres, *FEle* corneal front surface elevation at the thinnest point in relation to a 8 mm best-fit sphere, *BEle* corneal back surface elevation at the thinnest point in relation to a 8 mm best-fit sphere, *IHA* index of height asymmetry, *IHD* index of height decentration, *IOP* intraocular pressure, *ISV* index of surface variance, *IVA* index of vertical asymmetry, *Km* mean front keratometry, *Kmax* maximum keratometry, *m ± SD* mean ± standard deviation, *OI* osteogenesis imperfecta, *PPIavg* average pachymetric progression index

*Kolmogorov–Smirnov test

^#^Student t-test

**Fig. 1:**
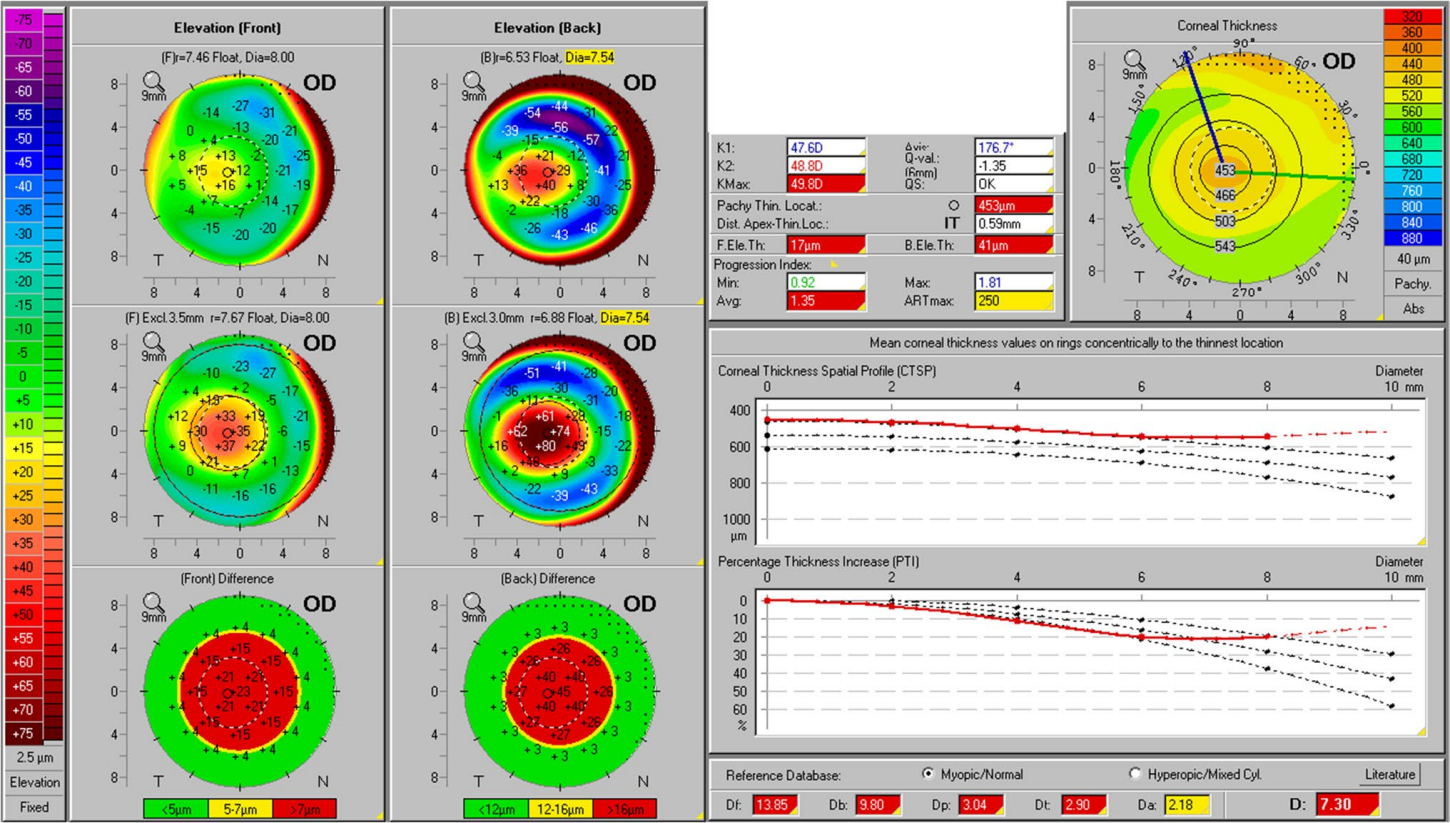
Belin-Ambrósio Enhanced Ectasia Display of the right eye of patient 9

**Fig. 2:**
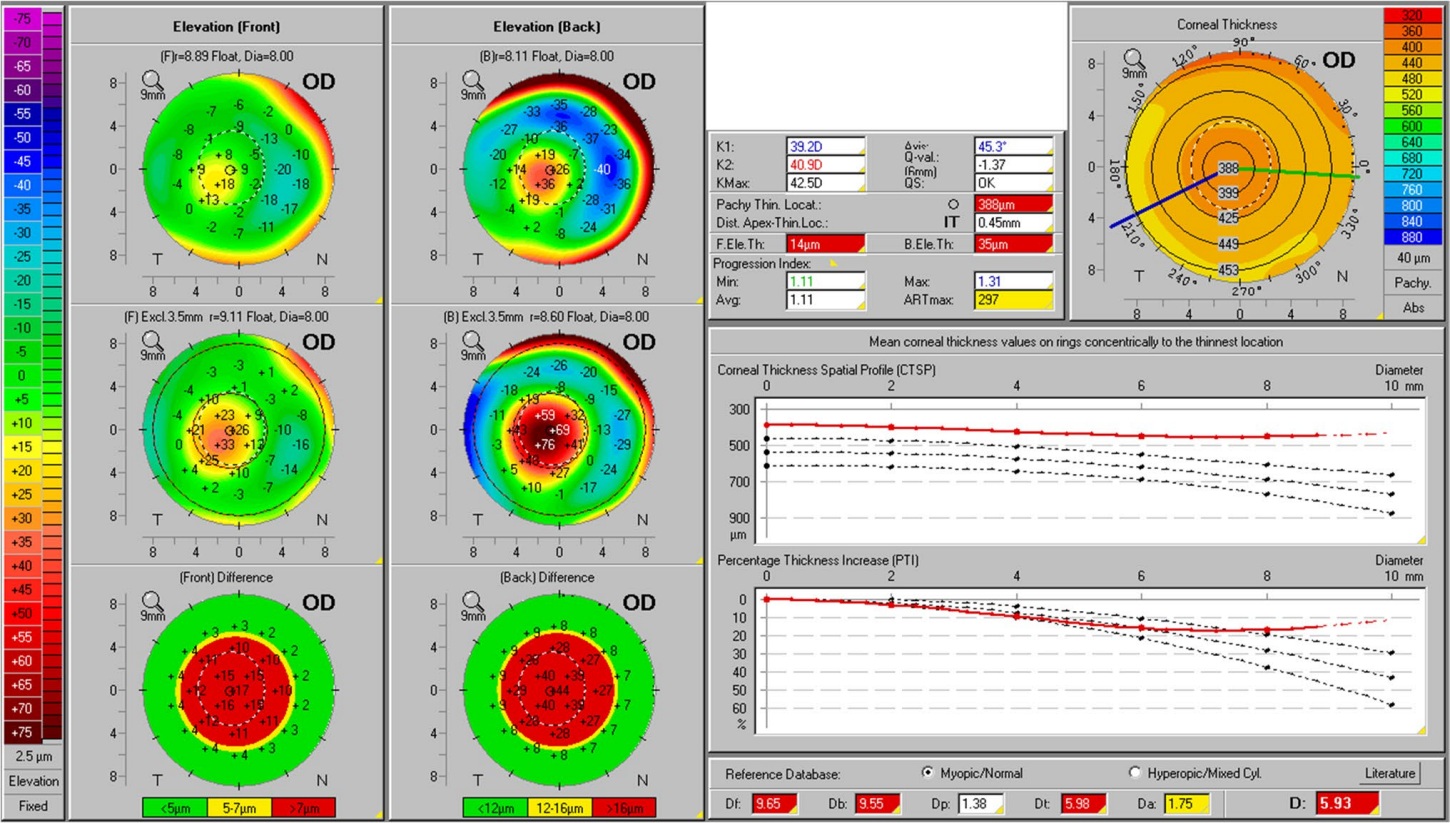
Belin-Ambrósio Enhanced Ectasia Display of the right eye of patient 14

When comparing the same indices of OI patients classified as type I with patients with other clinical forms, OI type I patients showed significantly lower thinnest corneal thickness, ARTmax and BAD-D (*p* < 0.005 for all comparisons; Table 2), with all remaining parameters being similar between both groups. The proportion of thinnest pachymetry < 500 µm was also significantly higher in OI type I patients versus other OI types (*n* = 41 vs. 8; 85.4% vs. 33.3%; *p* < 0.0001). Both patients with previously diagnosed bilateral KC showing overt clinical signs had OI type I disease (patients 9 and 14, Table 1). Both displayed nipple-shaped central cones (base < 5 mm and Kmax within the central 3 mm zone [40, 41]). Their inter-eye BAD-D average was 7.32 and 5.9, Km was 48.4 and 40.8D, Kmax 50.4 and 43.2D, PPIavg 1.5 and 1.1, ARTmax 216 and 275, front elevation 14.5 and 13.5 µm, back elevation was 43.5 and 33 µm, and minimum pachymetry was 456 and 390 µm. The right eye Belin-Ambrósio Enhanced Ectasia Display for both patients is shown in Figs. 1 and 2. We detected no other case of corneal ectasia with clinical signs at the slit-lamp.

**Fig. 3:**
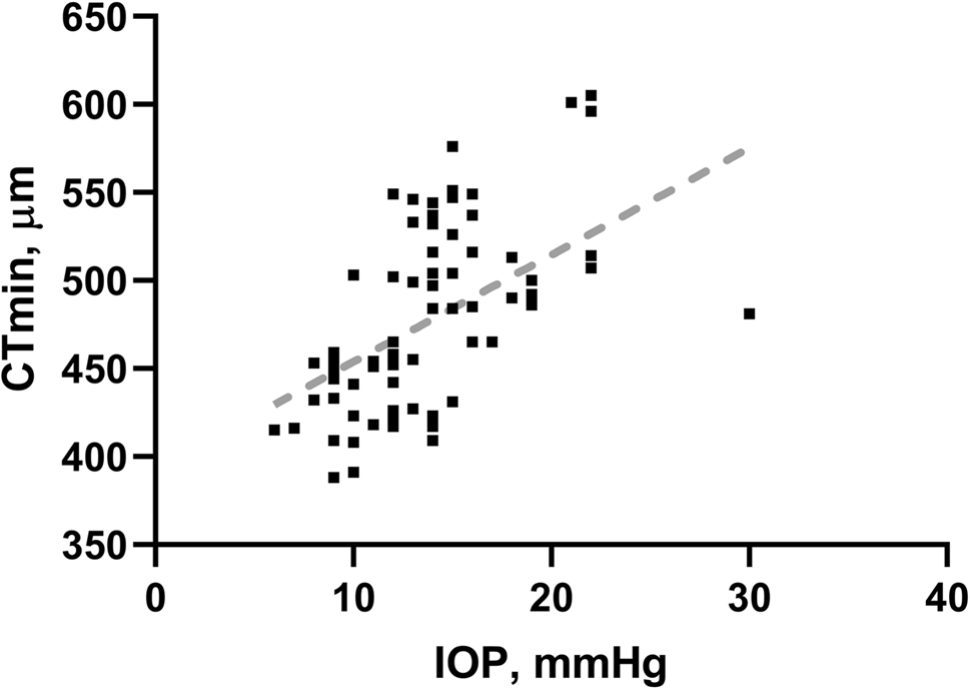
Best-fit line using linear regression model displaying the relationship between thinnest corneal thickness (CTmin) and intraocular pressure (IOP) in OI patients (squares)

IOP was significantly lower in OI patients versus controls (13.9 ± 4.6 vs. 16.5 ± 5.0 mmHg; *p* < 0.0001) and correlated with corneal thickness (r_s_ = 0.6226; *p* < 0.0001). A best-fit line using linear regression is showed in Fig. 3 to graphically display this relationship.

## Discussion

KC is a progressive corneal ectasia with a variably reported prevalence ranging from a worldwide 1.38:1000 prevalence to as high as 47:1000 in some populations, and is linked to allergy, asthma, eczema and eye rubbing behaviour [42, 43]. Corneal tomography is the methodology of choice for screening and early diagnosis of KC through the use of several indices [35–37]. The BAD-D index is commonly used in clinical practice and has been shown to be one of the most sensitive and specific indices in KC screening, with proposed cut-offs of 1.34–1.66 for suspect corneas and 2.38–2.69 for definite diagnosis [35–38].

Our sample included 74 eyes from 37 OI patients and the same number of control eyes and patients. Our results indicate a significant deviation from healthy subject readings on almost all parameters measured in OI patients. All these changes in OI patients skewed from normality towards readings associated with tomographic criteria used for ectasia diagnosis [35–37]. A recent study from Keles et al. comparing 17 OI patients with a control group showed a similar trend of tomographic changes in OI patients [20]. Arbitrarily considering BAD-D cut-offs of 1.6 and 2.4 for suspect corneas and definite keratoconus diagnosis, 47 eyes (64%) from 28 patients (9 unilateral) would be considered as having suspect corneas, and 22 eyes (30%) from 11 patients (2 unilateral) would be classified as having definite keratoconus, while in the healthy subject group no case was recorded with a BAD-D over these cut-off values. The proportion of eyes with a BAD-D over 1.6 and 2.4 in the OI type I subgroup was 71% (*n* = 34) and 35% (*n* = 17) versus 46% (*n* = 12) and 12% (*n* = 3) in the remaining OI clinical forms. This may be partly explained by the fact that BAD-D is a composite index representing an overall deviation from normality combining data from several tomographic parameters including elevation data, keratometry readings and pachymetric data. Approximately two-thirds of OI patients showed thin corneas < 500 µm compared with less than 3% of eyes in normal patients, in line with previously published reports [19, 20]. The thinner corneas in OI patients may skew the BAD-D to higher readings limiting its usefulness in screening for keratoconus in this population. Therefore, the authors believe BAD-D should not be used alone for this purpose in patients with OI, in agreement with other authors [24]. However, the clinical usefulness (or lack thereof) of this parameter has not been demonstrated in this patient population.

Our study also found that OI type I patients had significantly lower corneal thickness and higher proportion of blue sclerae than eyes of patients with other OI types. This is in line with findings by Evereklioglu et al. who determined a correlation between reduced corneal thickness and the presence of blue sclerae in OI patients, which is also much more prevalent in OI type I patients [8–10]. This may suggest that overall corneoscleral layer thickness more predominantly reduced in these. However, to the authors’ knowledge, there have been no published reports on scleral thickness in OI patients.

We also report lower IOP readings in OI patients compared to controls and a correlation between IOP in corneal thickness in these patients, in line with previous studies [9, 22, 32]. It has been well established that corneal thickness correlates with IOP [44]. Additionally, studies have reported lower corneal hysteresis and resistance factor in OI patients, with lower tonometry IOP readings but higher corneal-compensated IOP versus controls [22, 32]. Glaucomatous structural and functional damage has been linked to lower corneal thickness and hysteresis [45–47] and COL1A1 mutations have been directly linked to glaucoma cases [48]. These findings may indicate a higher susceptibility of OI eyes for the development of glaucoma and suggest that corneal biomechanics should be assessed in OI patients. Despite few reports of glaucoma cases in OI patients, no comprehensive study has studied glaucoma risk in this population [21, 22, 25, 26].

The main limitations of this study include its cross-sectional design, preventing from having multiple timepoint measurements, which would be useful in monitoring corneal changes over time. Moreover, this cohort consists of only Portuguese patients with known cases of familiarity, which may not represent the wider OI population.

To the authors’ knowledge, this is one of the largest OI samples in the literature describing corneal tomographic profiles. Both other large publications with similar design and purpose had significantly younger populations which may have limited their ability to diagnose ectasia [20, 24]. This is the first OI cohort study to detail the corneal profiles of 2 cases of definite bilateral KC in OI type I patients. In conclusion, we found significant corneal topometric, tomographic and pachymetric changes in OI patients compared with healthy subjects. A significant proportion of OI patients displayed tomographically suspect corneas, and two patients had bilateral keratoconus. Further studies are warranted to determine the risk of keratoconus in OI patients, such as longitudinal analysis. Additionally, efforts should be made to assess the clinical validity of currently available keratoconus diagnostic indices in collagen-deficient thin corneas, such as those found in OI patients.

## Data Availability

Data are available upon request.
